# Computational Analysis of the Effects of Fiber Deformation on the Microstructure and Permeability of Blood Oxygenator Bundles

**DOI:** 10.1007/s10439-024-03446-8

**Published:** 2024-02-13

**Authors:** Gianluca Poletti, Davide Ninarello, Giancarlo Pennati

**Affiliations:** LaBS - Department of Chemistry, Materials and Chemical Engineering “Giulio Natta”, Politecnico di Milano, Piazza Leonardo da Vinci 32, 20133 Milano, Italy

**Keywords:** Artificial lung, Hollow fiber membrane, Finite element model, Fluid dynamics, CFD, Bundle press-fitting, Pressure load

## Abstract

Mechanical loads on the polymeric fibers of oxygenating bundles are commonly present due to bundle press-fitting during device assembly and blood pressure load. However, computational fluid dynamics (CFD) simulations for fiber bundle optimization neglect possible changes in microstructure due to such deformations. The aim of this study is to investigate the impact of fiber deformability on bundle microstructure and fluid dynamics mainly in terms of permeability. Fibers from commercial mats typically used for blood oxygenators were mechanically tested and based on these experimental data, a material model was developed to simulate the structural deformations the fibers undergo under press-fitting and blood pressure loads. Then, CFD simulations were performed on deformed bundle repetitive units to investigate permeability under varying loading conditions. The effects of different bundle geometric parameters on the variation of bundle permeability due to press-fitting were evaluated. Bundle press-fitting results in significant changes in microstructure that are reflected in a bundle permeability more than halved for a 15% press-fitting. This impact on permeability is present in all the simulated fiber bundles and becomes more pronounced as the pitch between fibers and thus bundle porosity decreases. Instead, the analyses on pressurized bundle show only small deformations caused by pressure load, with permeability changes below 1%. While blood pressure effects could be neglected, bundle press-fitting turns out to have a significant impact on bundle microstructure and permeability. Neglecting such microstructure variations during CFD simulations could also lead to incorrect assessment of the local fluid dynamics within the bundle.

## Introduction

Blood oxygenators, also known as artificial lungs, are biomedical devices developed to temporarily replace or support the lung function. They are routinely used in cardiopulmonary bypass during open-heart surgery and for extracorporeal membrane oxygenation (ECMO) for patients with respiratory failure [16, 19]. Blood oxygenators exploiting hollow fiber membranes (HFM), arranged in bundle, have become a well-established solution over the years [16, 19]. To achieve blood oxygenation, such devices exploit hollow fibers, within which a gas flows, that are characterized by microporous walls permeable only to gas. During an extracorporeal circulation, the blood is forced to flow across the fiber bundle and specifically through the gaps between adjacent fibers allowing the gas exchange to occur. A fiber arrangement such that gas and blood are cross-flowing was identified as optimal condition for gas exchange [19]. Two main solutions to ensure cross flow were studied and carried out in the current commercially available devices: the stacked and wrapped configuration of the fiber bundle [13, 16]. In the stacked configuration, mats of parallel fibers are stacked on top of each other in the main direction of blood flow, with the fiber orientation rotated alternately 90° in each layer. Instead, in the wrapped configuration, the fiber mats are wrapped around a cylindrical core with the fibers angled with respect to the cylinder axis and positioned in such a way that adjacent fiber layers are alternately angled to each other. In this configuration, it is usual to have a choice of oxygenator inlet and outlet that forces the blood flow within the fiber bundle in axial, circumferential, or radial directions [1, 13].

Nowadays, Computational Fluid Dynamics (CFD) simulations are a useful tool to support the design of new blood oxygenators as they allow rapid evaluation of different design solutions, reducing the cost and time of iterative experimental research, and also provide virtual insights into local hemodynamic details within the fiber bundle [10, 20]. Due to the thousands of fibers within an oxygenator, the current approach for simulating the hemodynamics of the whole device is to describe the fiber bundle as a porous medium, using homogenized parameters without considering the bundle microstructure in detail [1, 7,10, 14, 21]. In fact, the homogenization of the fiber bundle microstructure requires the identification of equivalent parameters such as porosity and permeability of the porous medium. While porosity can be deduced analytically by knowing the geometry of the fiber bundle, the identification of permeability requires the definition of the relationship between the fluid superficial velocity and the pressure drop across the porous medium. Experimental permeability tests on the fiber bundle [1, 11] or on a scaled-up 3D-printed model [13] can be used to derive permeability values in the different flow directions. However, such experimental methodology could not be suitable for a rapid evaluation of the effects of geometric variations in the fiber bundle. Semi-empirical equations, such as the Ergun or Blake-Kozeny equation, that correlate permeability to fiber diameter and bundle porosity, were sometimes used in the literature for modeling the porous medium in blood oxygenator simulations [7, 14, 21]. However, these equations do not account for the precise fiber arrangement in the bundle and do not allow an evaluation of permeability anisotropy. By contrast, Low et al. [10] showed how CFD simulations that directly consider the fiber bundle microstructure modeling a repetitive elementary cell can be used for permeability estimation. This approach allows the variations in geometrical parameters to be easily assessed without the need for experimental tests on different fiber bundle prototypes.

In addition to the work of Low et al. [10], other models at the scale of single fibers [8, 15, 20] used to study local phenomena within the bundle, showed how the fluid dynamics can be influenced by fiber bundle microstructure. In these studies, geometrical aspects were evaluated such as fiber diameter and pitch between fibers [10], angle between fiber layers [20], and arrangement of the fiber layers varying from aligned, staggered, or random arrangements [8, 20]. However, the studied fiber bundle do not always represent commercial fiber bundle configurations but are often considered simplified arrangements that allow two-dimensional simulation of the domain [8–10, 15]. Despite the not negligible effect of local geometric aspects on the fluid dynamics, the contact between adjacent fiber layers is generally not modelled in microscale simulations and spaces between the fibers are artificially added to simplify the mesh creation [3, 5, 6, 8, 9, 15, 20]. These simplifications of the fiber bundle geometry in the CFD models are expected to affect the numerical estimation of bundle permeability and local fluid dynamics. Moreover, as oxygenating hollow fibers are commonly made of microporous polymeric materials [4, 16, 19], their interaction with each other and with the rigid housing could cause bundle deformation. Hence, the bundle microstructure could change as a result of the assembly within commercial blood oxygenators: compact fiber bundles are preferred to get more uniform fluid dynamics within the bundle [2], and a press-fitting of the bundle inside the oxygenator housing is commonly used in wrapped bundle configuration to prevent preferential shunt flow along the housing walls [1]. This aspect was partially modelled by Pelosi et al. [12], who accounted for the increased proximity between fiber centers given by the press-fitting of the fiber bundle in their microscale simulation. However, the deformation of the fiber bundle under the compressive loading due to press-fitting and the microstructure changes due to fiber-to-fiber interactions were not investigated, assuming a rigid movement of fibers and permitting fiber-to-fiber interpenetration. The polymeric fibers, despite being considered rigid in the totality of literature CFD studies, certainly experience deformations due to mechanical loads acting within the oxygenator: this occurs during the bundle assembly, if a press-fitting is applied, but can also arise as an effect of blood pressure during the oxygenator use, as suggested by Dipresa et al. [3] in their analysis of the current limitations of microscale models.

A correct description of the microstructure in presence of bundle deformation would allow a more accurate estimation of bundle permeability and local fluid dynamics. For this reason, the aim of this study is to investigate how fiber deformability affects bundle microstructure under press-fitting and blood pressure loads and what is the impact of microstructure changes on fluid dynamics. As a first step, the deformability of these structures was mechanically investigated with an ad hoc test, given the lack of experimental data on the mechanical behavior of the fibers in response to load conditions similar to press-fitting. Then, a material constitutive model was developed and used for finite element (FE) simulations of the structural deformation of the bundle. Finally, CFD periodic flow simulations on repetitive units, obtained as results of FE simulations, were set up for investigation of the impact of bundle deformations on local fluid dynamics, mainly in terms of bundle permeability.

## Materials and Methods

### Characterization and Modeling of Fiber Mechanical Properties

In order to have suitable mechanical data for calibrating the material model of the fibers, commercial hollow fibers used for oxygenator membranes were mechanically tested. Specifically, OXYPHAN PP 50/280 (3M Medical Membranes, Wuppertal, Germany) fibers were considered for the material characterization. These are Polypropylene fibers characterized by an outer diameter of 380 *µ*m and a wall thickness of 50 *µ*m, consistently with the material and size of hollow fibers commonly used in commercial blood oxygenators [4, 16].

**Figure 1:**
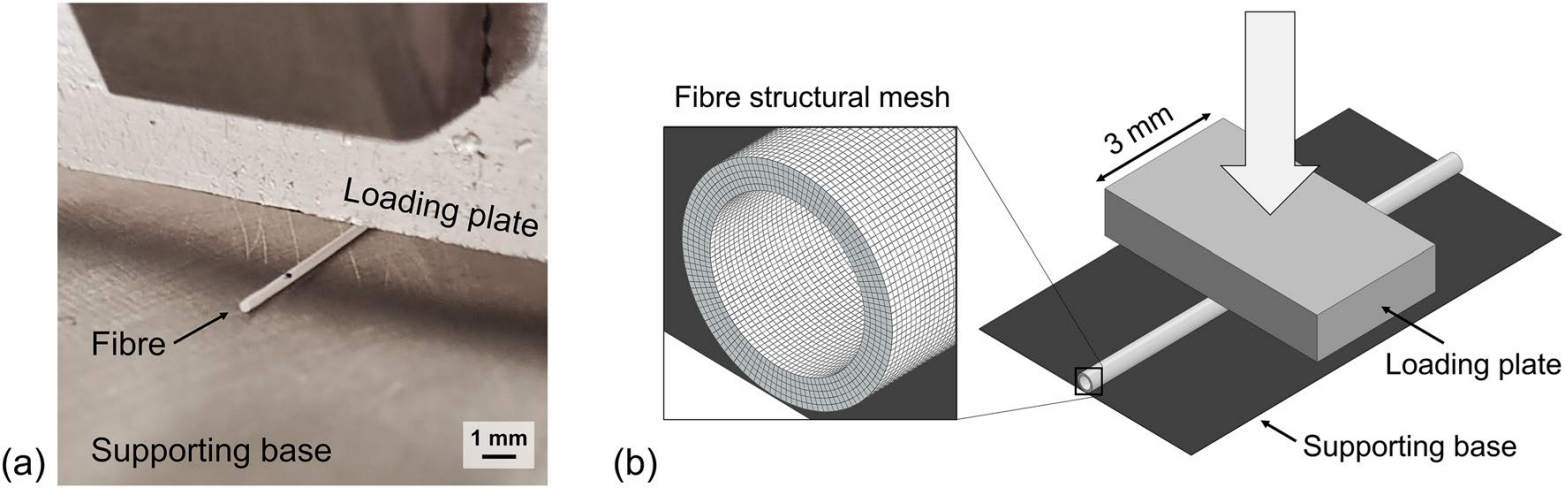
(a) Transverse compression testing of a single fiber for characterization of fiber mechanical behaviour; (b) Numerical replication of the experiment to calibrate the material model for fiber structural simulation: detail on the structural mesh used for fiber geometry discretization.

Due to bundle press-fitting inside the oxygenator housing, the bundle is compressed transversely to the fibers by the rigid walls. Consequently, it was decided to test the fiber stiffness directly in response to a transverse compressive loading (Fig. 1a). Namely, a rectangular plate with a width of 3 mm was adopted and the test was repeated on ten fiber samples to account for fibers variability. A preload was applied to the sample to guarantee flattening of the fibers onto the supporting base, as fibers can naturally bend. For each fiber, a displacement of the loading plate was applied to provide 15% macroscopic compression of the fiber at a rate of 10 *µ*m/s. For these mechanical tests, the BOSE Enduratec ELF 3200 mono-axial testing machine with a 20 N load cell was used.

The Force–Displacement results of the ten tests were recorded and averaged together. Since no reliable analytical formula is available for the identification of stress–strain behavior for such a mechanical test, the Force–Displacement data were directly used as a comparison term in the calibration of the material model by FE simulations. Based on the experimental results, the simplest hyperelastic model able to describe the mechanical behavior revealed by the tests was selected for the material modeling of the fibers. Therefore, a structural simulation replicating the experimental test was employed in the iterative process of identifying model parameters (Fig. 1b). Simulations were performed with the implicit solver of the commercial code ABAQUS (Dassault Systems Simulia Corp., Johnston, RI, USA) and the fiber geometry was discretized with hexahedral mesh elements of C3D8RH type. Accuracy in geometry discretization was ensured through a mesh sensitivity study aimed at identifying the optimal mesh element size (Fig. 1b). The experimental test was virtually replicated by simulating a fiber transversely compressed for a 3 mm portion by a rigid surface modeling the loading plate. The reaction force to plate displacement was analyzed and compared with the experimental data. A fine-tuning of the material model parameters was therefore used to best fit the experimental Force–Displacement data.

### Computational Analysis for Bundle Permeability Assessment

The performed computational analysis involved FE structural simulations to estimate the deformed fiber geometries under the occurring loads, followed by CFD simulations to study the effects of fiber deformations on the fluid dynamics inside the fiber bundle, and specifically on the bundle permeability. An oxygenator fiber bundle consists of thousands of fibers, but under appropriate assumptions and simplifications, repetitive units, representative of the microstructure, can be identified in the bundle geometry. Analysis can be performed on such repetitive units by setting proper boundary conditions without the need to simulate the whole bundle domain [5, 10].

**Figure 2:**
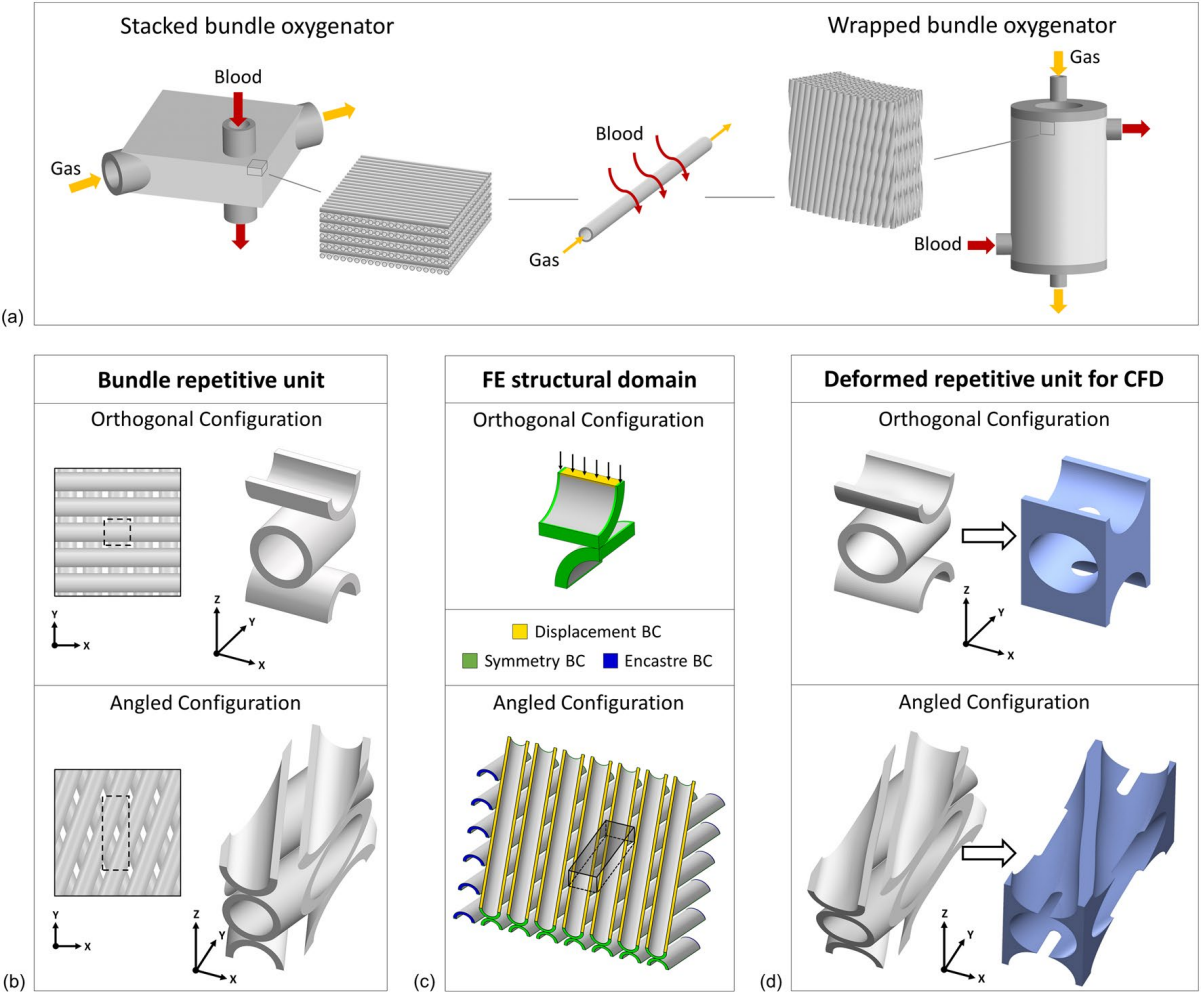
(a) Typical bundle arrangement in the stacked and wrapped bundle oxygenators and detail on gas and blood flow regions; (b) Fiber arrangement in the orthogonal and angled configuration and identification of the fiber bundle repetitive unit in the undeformed state i.e. in the absence of bundle press-fitting; (c) Structural domains considered for the simulation of bundle press-fitting and boundary conditions (BC) applied in the simulation for both orthogonal and angled configuration; (d) Deformed repetitive units of the bundle and of the fluid domain for CFD simulation.

Two different bundle configurations were analyzed in this study, an orthogonal and an angled arrangement of fiber layers. Typically, the former is used in stacked oxygenating bundles, while the latter is adopted in wrapped oxygenating bundles. Specifically, an angle of 20° with respect to the axial direction (Y-direction) was considered for the angled configuration (Fig. 2b). Although angled configurations are more typical of a wrapped bundle, the angled arrangement of fibers was studied in a Cartesian reference system without modeling a curvature of the bundle, as commonly done in the literature [13, 20]. Under the assumption of perfect fiber alignment and uniformity among the different layers constituting the bundle, repetitive units for both the bundle configurations were identified as shown in Fig. 2b.

Table 1 reports the investigated fiber bundles and the simulations performed to study the effects of mechanical loading. Three groups of analyses can be identified.

**Table 1:** The geometric parameters of the simulated bundles and the analyses performed on those bundles are reported for the three groups of simulations

Group of simulations	Simulated bundle	Bundle configuration	Fiber outer diameter [μm]	Fiber wall thickness [μm]	Fiber pitch [μm]	Porosity [−]	Structural analyses	CFD analyses
Reference bundles	O_380_50_500	Orthogonal	380	50	500	0.40	Press-fitting_FE; Press-fitting_interpenetrated; Pressurization	Permeability anisotropy
	A_380_50_500	Angled	380	50	500	0.40	Press-fitting_FE; Press-fitting_interpenetrated	Permeability anisotropy
Different fiber pitch (same fiber size)	O_380_50_450	Orthogonal	380	50	450	0.34	Press-fitting_FE	Permeability (main direction)
	O_380_50_550	Orthogonal	380	50	550	0.46	Press-fitting_FE	Permeability (main direction)
Different fiber size (same porosity)	O_200_25_263	Orthogonal	200	25	263	0.40	Press-fitting_FE	Permeability (main direction)
	O_300_25_395	Orthogonal	300	25	395	0.40	Press-fitting_FE	Permeability (main direction)

Press-fitting_FE: FE simulation of press-fitting, Press-fitting_interpenetrated: simplified press-fitting with allowed fiber-to-fiber interpenetration

First, an extended investigation was performed on both orthogonal and angled fiber configurations, for two reference bundles. The considered values for fiber diameter (380 *µ*m), wall thickness (50 *µ*m), and pitch between fibers (500 *µ*m) lead to a typical bundle porosity of 0.40. Namely, the impact of bundle press-fitting modeling (“Press-fitting”) on fluid dynamics was assessed considering different values of press-fit (from 0 to 15%). Moreover, the effect of blood pressure after bundle press-fitting was investigated to evaluate how the fiber transmural pressures (blood-gas pressure difference) acting during device usage change the inter-fibers geometry (“Pressurization,” only for bundle O_380_50_500). For the reference bundles, the permeability variations due to microstructure changes were evaluated in the different flow directions of interest investigating the permeability anisotropy.

Then, to extend the press-fitting analysis to other geometric parameters of the fiber bundle, two other sets of simulations involving different bundles were carried out (Table 1). For simplicity, only bundles with orthogonal configuration were evaluated and the results compared with the orthogonal reference bundle O_380_50_500. Moreover, only permeability in the main direction was analyzed.

A second set of simulations was used to analyze how the effect of press-fitting on fluid dynamics changes when the bundle porosity varied, while keeping the same fiber size. Considering the same fibers as the reference bundle, a variation of the pitch between fibers was set (450 and 550 *µ*m) to have a variation of the bundle porosity (0.34 and 0.46) (Table 1).

Finally, a set of simulations was designed to investigate how the effect of press-fitting on fluid dynamics changes when the fiber size varied, while keeping constant the bundle porosity. Specifically, based on information from the literature [4, 11] and commercial data of oxygenator fibers (3M Medical Membranes data sheets), fibers with outer diameter of 200 and 300 *µ*m were investigated in addition to the reference fibers (outer diameter of 380 *µ*m). These smaller fibers show a reduced wall thickness (25 *µ*m) in accordance with commercial fibers. To have the same porosity in the different bundles and equal to the porosity of 0.40 of the reference bundle O_380_50_500, a pitch of 263 *µ*m and a pitch of 395 *µ*m were selected for the diameter of 200 and 300 *µ*m, respectively (Table 1).

#### Finite Element Structural Analysis

During fiber bundle press-fitting, the load of the housing walls onto the bundle external fibers creates a fiber-to-fiber compression between the inner fibers of the bundle. Under the assumption that a macroscopic bundle press-fitting would lead to a uniform fiber deformation across the bundle, it was decided to model the press-fitting at the scale of the repetitive units considering Z-direction as the direction of load application. Once a material model able to correctly describe the fiber mechanical behavior was defined, this model was used to virtually investigate this kind of fiber deformation. Specifically, for the effects on bundle assembling, three different levels of bundle press-fitting were simulated corresponding to 5%, 10%, and 15% compression of the bundle inside the oxygenator housing (Press-fitting_FE in Table 1).

To properly model the boundary conditions of the repetitive units of press-fitted bundles, it was necessary to consider fiber domains for the FE simulation slightly different from the geometry of the repetitive unit itself. Two different methodologies were exploited to simulate the fiber deformation depending on whether to consider an orthogonal or an angled configuration of the bundle (Fig. 2c). Specifically, accounting for the symmetries in the repetitive unit of the orthogonal configuration, a domain for the structural simulation was identified for this configuration corresponding in one-eighth of the bundle repetitive unit. Appropriate symmetry boundary conditions were set on the faces of the structural domain to represent a portion of the fiber bundle far from the housing walls. In order to replicate the bundle press-fitting, a displacement boundary condition on the top face of the upper fiber and a symmetry condition on bottom face were set in Z-direction (Fig. 2c). Instead, for the angled configuration, due to the lack of structure symmetries in the XY plane and the computational cost of setting periodic boundary conditions in the structural simulation, an extended domain of fibers was simulated such that a central portion involving more than one repetitive unit was not affected by unrealistic boundary conditions of symmetry and encastre.

Once the deformed geometry of the simulated domain was obtained, the bundle repetitive unit at the different degrees of press-fitting was derived (Fig. 2d). Specifically, for the orthogonal configuration the simulated one-eighth of repetitive unit was mirrored to get the whole unit, while for the angled configuration the repetitive unit was extracted from the simulated domain. Starting from the bundle repetitive units the fluid repetitive units were derived for the CFD simulations to analyze the impact of bundle press-fitting on the fluid dynamics. FE simulation of press-fitting followed by CFD analysis was performed for all the bundle considered in this study as shown in Table 1.

To investigate the importance of considering local deformations, as derived from the structurally simulated press-fitting, in the geometries for the CFD simulations, a simplified approach accounting for fiber-to-fiber interpenetration was considered for the sake of comparison. Namely, a rigid movement of fibers allowing to account for the reduced distance between fibers and approximate the press-fitting without involving a structural simulation was applied. Therefore, for the orthogonal and the angled reference bundles, press-fitted repetitive units were reproduced at the three analyzed compression degrees (5%, 10%, and 15%) using the interpenetrated fibers approach (Press-fitting_interpenetrated in Table 1).

For the pressurization of the orthogonal reference bundle O_380_50_500, a pressure load was applied to the outer surface of the fibers equivalent to the pressure difference across the fiber walls between the blood and gas during the device use. The gas flowing inside the hollow fiber is usually at atmospheric pressure, while the blood has a pressure value around 200 mmHg above the atmospheric level accounting for the patient pressure and the pressure drop downstream of the fiber bundle [17]. This pressure was then applied to the fibers both as the first load acting on an undeformed geometry corresponding to a zero press-fitting condition and as the second load acting following the bundle press-fitting.

Furthermore, for all simulated cases, the bundle porosity was calculated at the different degrees of press-fitting as the ratio of the fluid volume of the repetitive unit (resulting from the deformed fiber geometry) over the volume of the box having nominal dimensions of the repetitive unit.

#### Computational Fluid Dynamics Analysis

For the investigation of bundle permeability, periodic flow simulations on the repetitive units were used [5, 10]. Such simulations allow the simulated repetitive cell to have flow conditions representative of the inner portion of the fiber bundle far from boundary effects. Considering that the region of the bundle far from boundary effects is a predominant portion of the bundle, the permeability estimated at the level of the repetitive unit could be considered representative of the bundle.

**Figure 3:**
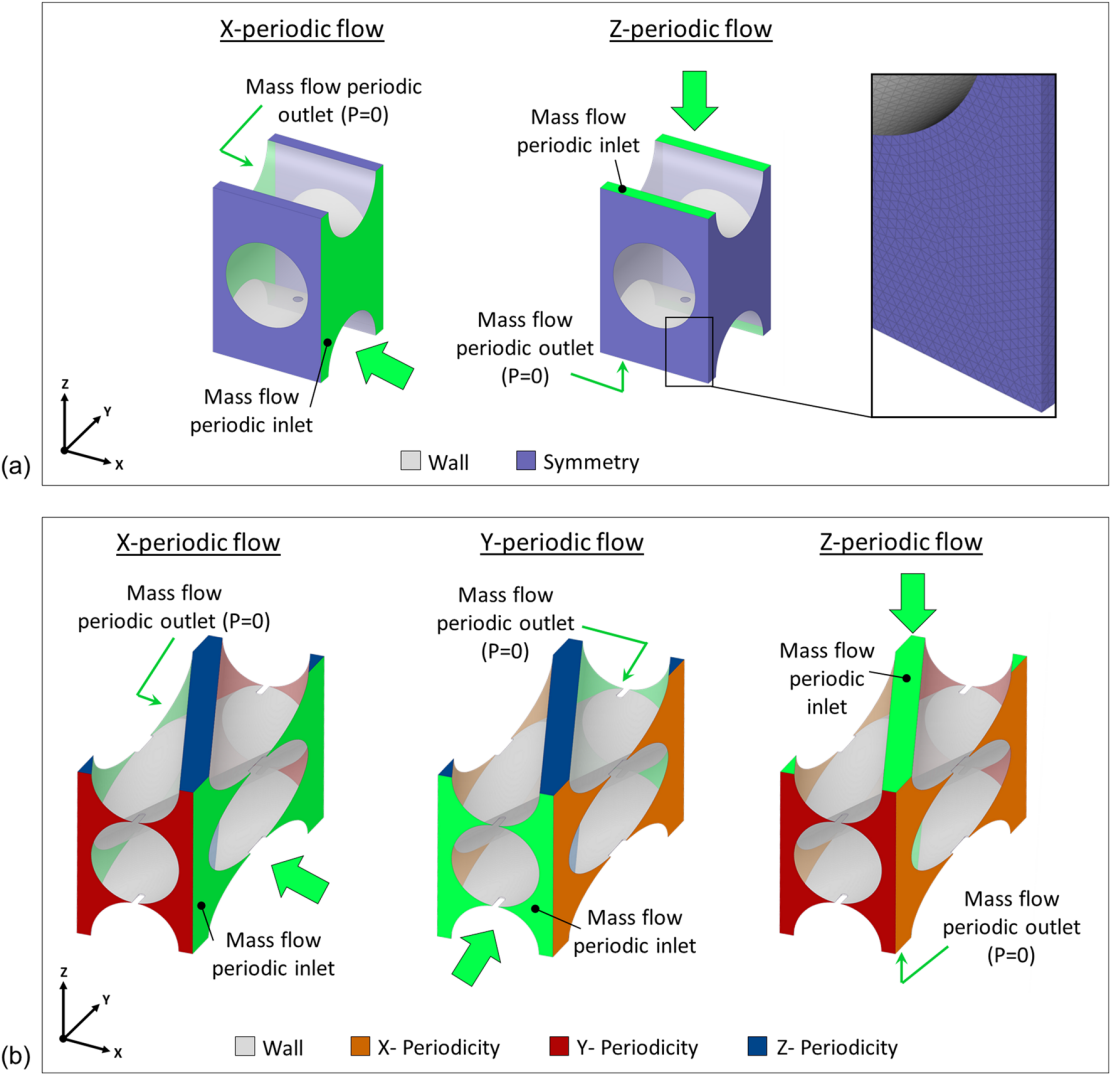
For both the orthogonal configuration (a) and the angled configuration; (b) the different flow directions tested for the anisotropy investigation and the boundary conditions set in the periodic flow simulations are shown (fiber walls are illustrated in transparency to allow visualization of all faces). In addition, a detail on the mesh used to discretize the fluid volume is shown for the orthogonal configuration.

Repetitive units of fluid domain were derived from the bundle repetitive units by a Boolean operation of subtracting the fibers and the volume inside them from a box having nominal dimensions of the repetitive units (Fig. 2d). During the generation of the fluid geometry, the contact area between the fibers was carefully managed where infinitesimal volumes occur around the contact area. This is mainly a problem for mesh generation, and it is common in the literature to fictitiously space fibers tens of micrometers apart [5, 8, 9, 20]. As in this study the contact between the fibers was intended to be involved, it was decided to slightly extend the contact area so as to ensure a thickness of at least 1 *µ*m in the fluid volume. The resulting geometries were discretized in finite volumes for the CFD simulation within the commercial software ANSYS® Fluent 2021 R2 (ANSYS Inc., Canonsburg, PA, USA). Tetrahedra were identified as the element type best suited to discretize the deformed repetitive unit. To ensure the independence of the solution from the mesh, a mesh sensitivity analysis was carried out simulating the fluid dynamics with different meshes characterized by different element sizes. Specifically, meshes obtained by doubling the number of volumetric elements time by time were compared with each other. The mesh that ensured a difference with respect to the finest mesh of less than 1% in terms of pressure drop across the cell was considered as the optimal mesh. The mesh found to satisfy this requirement has about 0.7 million volumetric elements for the orthogonal configuration and 1.1 million volumetric elements for the angled configuration with a surface mesh characterized by a minimum size of 0.0025 mm and a maximum size of 0.01 mm (Fig. 3a).

To investigate the anisotropies in the permeability of both an orthogonal and angled bundle, periodic flow simulations were conducted on the units of reference bundles in the different directions of interest (Fig. 3). Specifically, for the periodic flow simulation, a defined value of mass flow rate was set at the inlet and outlet faces of the periodic unit with direction normal to the periodic faces and a boundary condition of pressure equal to 0 was imposed at the periodic outlet face. Regarding the orthogonal fiber arrangement, typical of a stacked bundle configuration, the Z-direction is the main flow direction in a stacked bundle, and the X- and Y-directions are transversal flow directions with the same microstructure in those directions and were therefore treated as one and the same transversal flow direction (X-direction) [13]. In an orthogonal fiber arrangement, the faces bounding the repetitive unit are also symmetry faces, and for this reason symmetry boundary conditions were set on those faces when not investigating the periodic flow across the face [5] (Fig. 3a). Wall boundary conditions were instead applied to the surfaces corresponding to the outer wall of the fibers. Regarding the angled fiber arrangement, which could be seen as representing a wrapped bundle, the microstructure is different in the X-, Y-, and Z-directions (hypothetically corresponding to the circumferential, axial, and radial directions of a cylindrical bundle). Therefore, periodic flows were set up time by time in the different directions to identify the corresponding permeability (Fig. 3b) [13]. In an angled fiber arrangement, the faces of the repetitive unit are not symmetry faces, so it was required to set periodic boundary conditions even when not directly investigating the flow across the face. While a detailed analysis of the permeability in the different flow directions was performed for the reference bundles, for simplicity, for the other orthogonal bundles involved in this work only the permeability in the main flow direction (Z-direction) was evaluated for the comparison with the orthogonal reference bundle.

As interested in evaluating the permeability, which is a property of geometry and independent of fluid, simulations were carried out with water as fluid [10, 13]. In the different units and flow directions simulated, the mass flow rate at the inlet of the repetitive units was set such that the permeability was evaluated in a correct range of velocities. Specifically, fluid dynamics conditions were simulated with a Reynolds number equal to 1, representative of the operating conditions of an oxygenator [10, 15, 16, 20]. Preliminary simulations (not reported results) demonstrate a good linearity in the relationship between flow rate and pressure drop across the repetitive unit indicating a correct application of Darcy's law and the possibility of deducing permeability from a single value of flow rate [10, 13]. Specifically, by giving as input a flow rate $Q$ in the periodic flow simulation, the pressure drop $\Delta P$ was obtained and used for the calculation of the permeability $K$ using the Darcy's law according to the following formula:

$$K=\upmu \frac{Q}{A} \frac{L}{\Delta P},$$

where $\upmu$ is the fluid viscosity (1 × 10^−3^ $\frac{Kg}{m s}$ for water), $A$ is the cross section of the porous medium associated to the repetitive cell with Darcy’s law, and $L$ is the length of the cell in the analyzed flow direction. For each bundle, different values of $A$ and $L$ were considered depending on the flow direction and degree of press-fitting. Furthermore, when analyzing the permeability variation at different degrees of press-fitting, the permeability values were normalized with respect to the permeability value estimated in the absence of press-fitting (${K}_{0}$).

To study the effect of fiber pressurization on the microstructure, periodic flow simulations were also carried out on the pressurized repetitive units of the O_380_50_500 bundle and compared with the corresponding no-pressurized units. The comparison was made in terms of permeability, and the percentage error committed in the permeability estimation by neglecting pressurization in the determination of microstructure was evaluated.

The ANSYS® Fluent 2021 R2 software was used to solve fluid dynamics in a steady-state simulation with laminar flow model. Residual thresholds of 1 × 10^−5^ for continuity, velocity components, and pressure drop across the repetitive unit were set for the evaluation of solution convergence.

## Results

**Figure 4:**
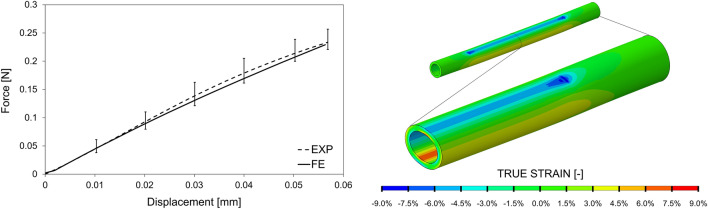
On the left, average Force–Displacement experimental curve (EXP) with associated range of variability related to the 10 compression tests and computational curve (FE) resulting from the material model calibration to best fit the experimental curve. On the right, fiber strain map of the numerical replication of transverse compression with detail on half of the fiber compressed by the loading plate.

The Force–Displacement data corresponding to the transverse compression of 10 single fiber samples were averaged together, and the average curve with value variability range is shown in Fig. 4. A nearly linear relationship between force and displacement is observed in the values tested. This curve was exploited for the calibration of a material model to be used in structural simulation of fiber deformation. Based on the experimental data, a Neo–Hookean hyperelastic model was chosen as the material model for numerical simulations. Neo-Hookean strain energy potential ($U$) is described by the following formula:

$$U={C}_{10}\left(\overline{{I }_{1}}-3\right)+ \frac{1}{{D}_{1}} {\left({J}_{el}-1\right)}^{2},$$

where $\overline{{I }_{1}}$ is the first strain invariant, ${J}_{el}$ is the elastic volume strain, and ${C}_{10}$ and ${D}_{1}$ are the model parameters to be calibrated. Through an iterative process of variation of model parameter values in the numerical simulation of the fiber compression, ${C}_{10}$ equal to 17.308 MPa and ${D}_{1}$ equal to 0.027 MPa^−1^ were identified as the parameters that best capture the experimental curve (Fig. 4).

**Figure 5:**
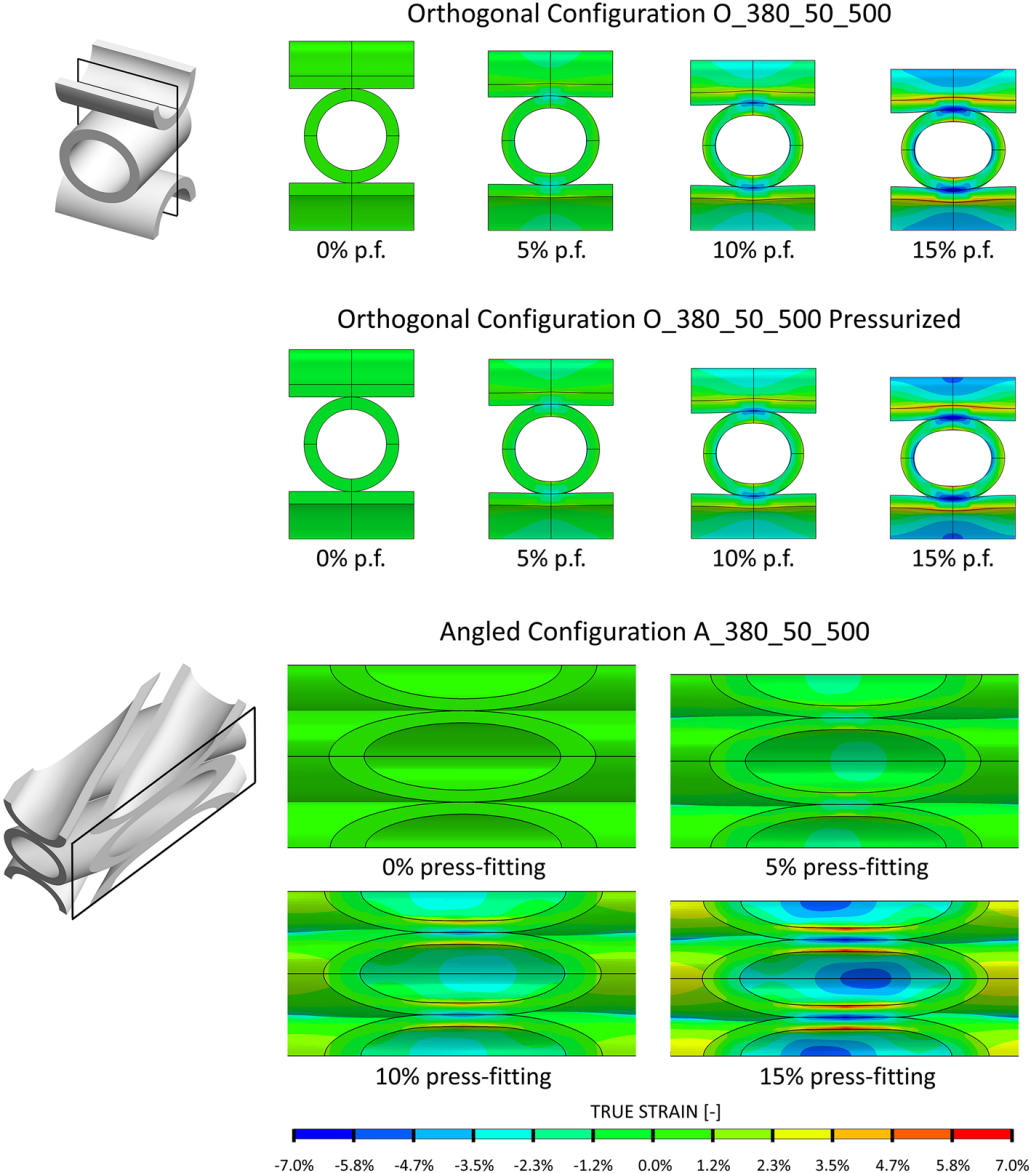
True strain visualization of the bundle repetitive unit at the different levels of press-fitting (p.f.) simulated for the orthogonal configuration (O_380_50_500) without and with further pressurization step and for the angled configuration (A_380_50_500). For visualization purposes, the simulated structural domain was mirrored/cut to show the strains at the level of repetitive units.

The results in terms of fiber strains at different degrees of press-fitting, obtained from the structural simulations performed using this material, are shown in Fig. 5 for the reference bundles. Negative and positive strain values are observed with the fiber wall mainly subject to bending stresses as a consequence of bundle press-fitting. As the press-fitting degree increases, there is an ever more pronounced lateral widening of the fiber cross section, which changes from circular to elliptical shape in the compression zone. The deformed geometries of the other simulated bundles are not reported, but a similar distribution of deformations was noted.

Figure 5 also reports the deformed geometries of bundle O_380_50_500 as a result of pressure loading applied after press-fitting. The external pressurization of the fibers given by blood appears not to alter the deformed geometry of the fibers, as negligible variations in local strains at the different degrees of press-fitting occur with the addition of a pressurization step.

The variation in bundle porosity resulting from the press-fitting process was calculated for the analyzed bundles and reported in Table 2. A similar variation of porosity as the degree of press-fitting changes is observed for all bundles with the same original porosity despite having different bundle configurations and different fiber sizes. Specifically, starting with a 0% press-fitting porosity of 0.4, these bundles reach porosity values between 0.31 and 0.32 at 15% press-fitting. Similarly, a reduction in porosity is also observed for bundles with a different original porosities, with porosity values going from 0% press-fitting values of 0.34 and 0.46 to values of 0.23 to 0.37 at 15% press-fitting, for bundles O_380_50_450 and O_380_50_550, respectively.

**Table 2:** Bundle porosity variation due to bundle press-fitting for the different simulated cases.In addition to the bundles whose press-fitting was modelled with structural simulation, the results of press-fitting with an interpenetrated fibers approach are also reported for bundles O_380_50_500 and A_380_50_500

	Bundle porosity [−]			
	0% Press-fitting	5% Press-fitting	10% Press-fitting	15% Press-fitting
0_380_50_500	0.40	0.37	0.34	0.31
0_380_50_500_interpenetrated	0.40	0.38	0.35	0.32
A_380_50_500	0.40	0.37	0.34	0.31
A_380_50_500_interpenetrated	0.40	0.37	0.35	0.32
0_200_25_263	0.40	0.37	0.35	0.31
O 300 25 395	0.40	0.38	0.34	0.31
0_380_50_450	0.34	0.31	0.27	0.23
0_380_50_550	0.46	0.43	0.40	0.37

**Figure 6:**
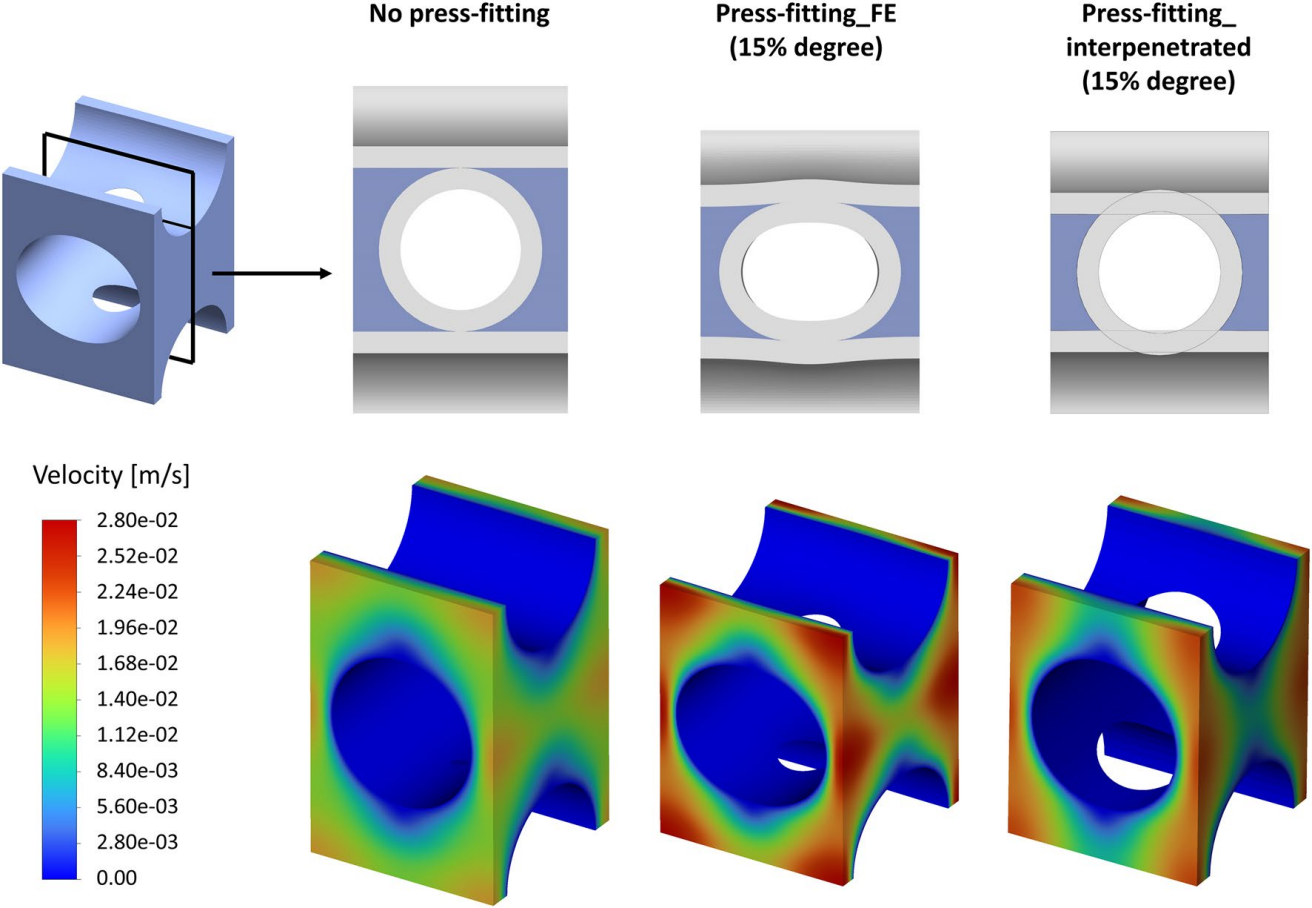
On the top, fiber and fluid geometry in the cross section (highlighted by the plane) of the orthogonal repetitive unit for the cases of no press-fitting, Press-fitting_FE (15% degree), and Press-fitting_interpenetrated (15% degree) is reported. On the bottom, the velocity maps resulting from a Z-direction periodic flow simulation are shown.

A comparison of the press-fitted geometry derived from the Press-fitting_FE and Press-fitting_interpenetrated approaches is shown in Fig. 6 for the reference orthogonal bundle. As can be observed, the simplified approach does not allow the lateral widening of the fiber cross section to be captured. However, very similar porosity values are noted when compared the simplified geometries with the structurally deformed geometries at different press-fitting levels and for both the orthogonal and angled bundle configurations (Table 2).

Figure 6 shows the velocity map results of CFD periodic flow simulations for the orthogonal bundle in case of no press-fitting and 15% press-fitting with both the approaches mentioned above. A relevant increase in velocities is observed when comparing the fluid dynamics of the unit subjected to a 15% press-fitting versus the unit in the absence of press-fitting. Instead, when comparing the two press-fitting modeling approaches, a similar velocity map is noticed despite an underestimation of 19% of the maximum velocity by the interpenetrated fibers approach.

**Figure 7:**
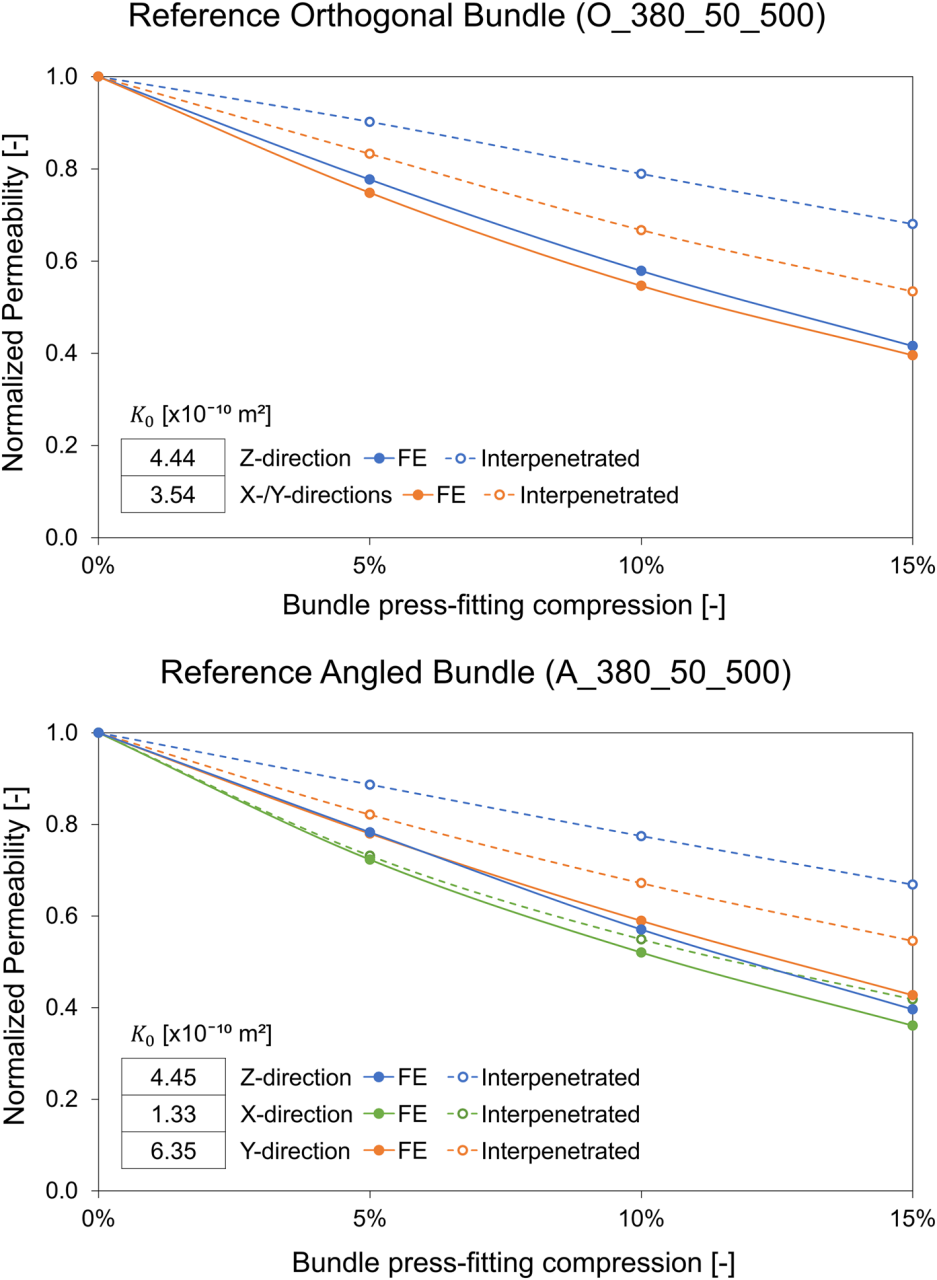
Trend of the permeability normalized on ${K}_{0}$ at the different simulated degrees of press-fitting for the orthogonal configuration (top) and angled configuration (bottom) of the reference fiber bundles. Permeability values in the different flow directions of interest are shown for press-fitted geometries estimated by both Press-Fitting_FE and Press-Fitting_interpenetrated approaches. The permeability value ${K}_{0}$ of the 0% press-fitted bundle is reported for each analyzed direction in the table within the graph.

CFD simulations were used to estimate the permeability of the orthogonal and angled reference bundles in the different flow directions of interest. Anisotropy in the permeability of the fiber bundle is observed in absence of press-fitting (Fig. 7). Specifically, anisotropy seems to be less remarkable for the orthogonal bundle, with permeability values ${K}_{0}$ varying from 4.44 × 10^−10^ (Z-direction) to 3.54 × 10^−10^ m^2^ (X-direction), and more pronounced for the angled bundle, with ${K}_{0}$ equal to 1.33 × 10^−10^, 6.35 × 10^−10^, and 4.45 × 10^−10^ m^2^ in the X-direction, Y-direction, and Z-direction directions, respectively.

The variation of permeability normalized over ${K}_{0}$ values as a function of the degree of press-fitting is shown in Fig. 7. With regard to the FE simulated press-fitting, for both orthogonal and angled bundles, a similar reduction in normalized permeability is observed in all the tested flow directions as the degree of press-fitting increases, confirming an important effect of this variable. Specifically, the progressive decrease of the normalized permeability leads down to values in the range of 0.4 at a press-fitting level of 15%, thus indicating a more than halved permeability compared to the ${K}_{0}$ value of permeability in the absence of press-fitting. The trends are quite similar in the different flow directions, indicating that the observed anisotropy remains with bundle press-fitting.

The trend of permeability reduction as press-fitting increases for the geometries derived from the Press-fitting_interpenetrated approach is also shown in Fig. 7. Permeability estimation errors with respect to the Press-fitting_FE approach are found to always be overestimation errors increasing with the degree of press-fitting. In contrast to what observed for structurally deformed geometries, different levels of permeability reduction are estimated in the different flow directions analyzed resulting in a permeability estimation error varying with the flow direction. Specifically, the highest errors (over 60% at a press-fitting level of 15%) are found in the Z-direction of both bundles, while the lowest errors are observed in the X-direction of the angled bundle.

The permeability of the pressurized repetitive unit O_380_50_500 was deduced by CFD simulation and compared with the values estimated in the absence of bundle pressurization at the different degrees of simulated press-fitting. Permeability differences of less than 1% were observed, in agreement with the small changes in the deformed geometry shown in Fig. 5.

**Figure 8:**
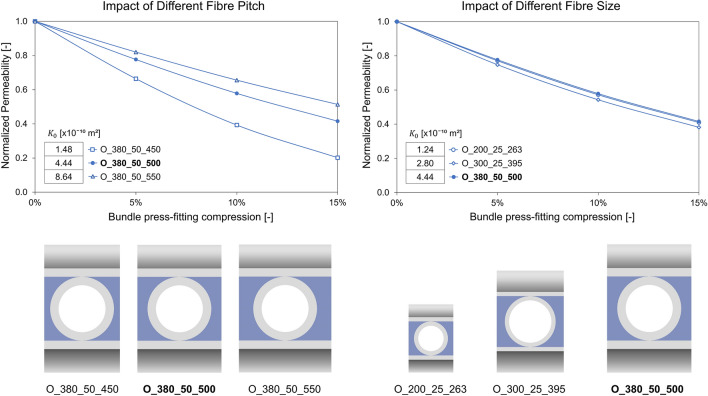
Comparison of the different simulated bundles for the study of deformation impact in the presence of different fiber pitches (left) and fiber sizes (right): on top the trend of the permeability normalized on ${K}_{0}$ evaluated in the Z-direction at the different simulated degrees of press-fitting and on the bottom the geometries of the bundles involved in the comparison (both fiber and fluid domain are reported together). The permeability value ${K}_{0}$ of the 0% press-fitted bundle is reported for each analyzed bundle in the table within the graph.

Finally, the results of the study for the analysis of the impact of fiber pitch (related to bundle porosity) and fiber size on the reduction of permeability due to bundle press-fitting are shown in Fig. 8. Specifically, the trend of the permeability normalized on ${K}_{0}$ at the different simulated press-fitting degrees, evaluated in the main flow direction (Z-direction) of the analyzed bundles, is reported. Variation in fiber pitch with the same fiber size appears to have a relevant impact both in terms of variation in the value of ${K}_{0}$ observed in different bundles and in trend of the normalized permeability as press-fitting changes. Furthermore, despite an equal increase and decrease in pitch (± 50 *µ*m) compared to the reference value of 500 *µ*m, a much more significant impact on the variation of normalized permeability is observed in the case of pitch reduction (O_380_50_450) with a normalized permeability value of 0.2 at a press-fitting degree of 15%, approximately halved compared to the case of reference bundle O_380_50_500 and reduced by a factor of 5 compared to the permeability ${K}_{0}$ in absence of press-fitting. When comparing different fiber sizes with the same bundle porosity, a significant decrease in permeability ${K}_{0}$ is observed as the fiber size decreases. However, despite the different ranges of permeability values, once the normalized permeability is compared, a very similar reduction is observed as the degree of press-fitting increases, suggesting a comparable impact of press-fitting on bundle permeability in all the simulated fiber sizes.

## Discussion

The fiber bundle in a blood oxygenator consists of thousands of polymeric microporous fibers that can deform when subjected to external loads. However, to the best of our knowledge, there is always the common practice in the literature of neglecting such fiber mechanical deformations when computational simulations are involved in the evaluation and optimization phase of the oxygenator and its bundle microstructure. To evaluate the feasibility of this assumption, in this study, structural simulations of fiber press-fitting and pressurization were performed, and the estimated deformed geometries were used to perform CFD analyses. A realistic fiber deformability was included, as ad hoc experimental tests allowed us to characterize the mechanical behavior of commercial fibers and calibrate a suitable material model. During the press-fitting phase, the fiber bundle experiences a transverse compression given by the housing walls, which results in localized fiber-to-fiber compression involving bending stresses in the fiber wall. Given this type of loading, it was decided to test the mechanical behavior of the fibers in response to a transverse compression load and through an iterative process of numerical replication of the experimental test the material model was calibrated. A simple hyperelastic constitutive model such as the neo-Hookean model is sufficient to well capture the experimental behavior, considering the observed data variability. A linearity in the mechanical behavior of the material at such low range of strain values, and the observed stiffness values are in agreement with the few data available in the literature, concerning, however, tensile testing of Polypropylene fibers for non-biomedical applications [18].

For the computational analysis, some assumptions and simplifications were required. The bundle microstructure and permeability were investigated at the level of a repetitive unit under the assumption that the unit is representative of a major bundle portion and that external loads acting on the bundle are transferred uniformly within the bundle. This repetitive unit is based on the simplification of neglecting the presence of irregular position of fibers within the bundle, however, it could represent an average microstructure of the random fiber positions. In this work, it was decided to study both an orthogonal and an angled configuration of the fiber bundle. While for an orthogonal configuration the repetitive unit except for irregularities in the bundle is well representative of a stacked bundle, in the case of an angled bundle configuration, of greater interest for a wrapped bundle oxygenator, the repetitive unit is defined in a Cartesian reference system not formally representing a cylindrical bundle. This simplification arises from the impossibility of identifying a reasonable repetitive unit of a wrapped bundle in which periodic flow simulations can be performed. Indeed, macroscopic domain of the fiber bundle should be simulated with related computational cost issues. Finally, for the simulation of press-fitting, structural domains different from the repetitive units were considered, but by means of appropriate boundary conditions, it was possible to obtain a correct simulation of a repetitive domain representative of the central portion of the bundle away from boundary effects.

In-depth permeability analyses were carried out on two reference bundles with orthogonal and angled configurations numerically investigating the anisotropy in the bundle permeability, in addition to a simple attempt to evaluate the permeability changes with other bundle parameters such as porosity and fiber size. Although it is difficult to individually compare the estimated permeability values with literature values, given the high sensitivity of permeability to variations in the studied bundle microstructure, the permeability values calculated in the absence of press-fitting turn out to be in the literature range of permeability values of 1.8–16.1 × 10^−10^ m^2^, corresponding to literature works considering bundles similar to those involved in this study [7, 10, 13]. Within this range of values, the obtained permeability values are consistent with the detected dependence of permeability on bundle parameters and a similar behavior in the permeability anisotropy is observed. Specifically, the results show for an orthogonal bundle configuration a higher permeability in the main direction than in the transverse direction of a stacked bundle, and for the angled configuration a maximum permeability in the axial direction and minimum in the circumferential direction of a hypothetical cylindrical bundle, in agreement with experimental permeability tests [13].

To investigate the impact of press-fitting on the bundle microstructure and fluid dynamics, the permeability was evaluated as the degree of press-fitting varied. The results of the study show a significant impact of press-fitting with permeability values less than 80% of ${K}_{0}$ at a 5% press-fitting condition and about 40% of ${K}_{0}$ at a 15% press-fitting. These results are valid for both the studied orthogonal and angled configuration, for all the analyzed flow directions and for the three commercial fiber diameters of 200, 300, and 380 *µ*m with a bundle porosity of 0.40. Indeed, although different fiber diameters and wall thicknesses lead to different values of fiber mechanical stiffness, the impact of press-fitting was found to be similar. Instead, the bundle porosity and especially the pitch between the fibers prove to be parameters with a relevant effect on the press-fitting impact on the bundle permeability. In fact, as the pitch between the fibers decreases, an ever more significant reduction in permeability is observed when press-fitting is applied to the bundle. The analysis of the O_380_50_450 bundle, involving a 50 *µ*m pitch reduction with respect to the reference case, shows that a bundle press-fitting of 15% leads to a permeability value equal to about only 20% of ${K}_{0}$. The greater sensitivity to press-fitting of a bundle with lower porosity can be explained by the fact that the lateral widening of the fiber elliptical cross section given by press-fitting has a greater weight on the gap between the fibers in the presence of a reduced pitch. For the same reason, the press-fitting has a lower impact on permeability, although still significant, in the presence of increased pitch in the fiber bundle.

This analysis also enabled an estimation of the errors committed by the literature approach of neglecting the bundle press-fitting when modeling the bundle microstructure for a CFD simulation. Specifically, neglecting a 5% press-fitting leads to permeability estimation errors in the range of 20%, 30%, and 50% for bundles with porosity of 0.46, 0.40, and 0.34, respectively. Such errors increase with the press-fitting degree until neglecting a 15% press-fitting results in the estimation of permeability values 2, 2.5, and 5 times higher (for 0.46, 0.40, and 0.34 bundle porosity). In addition, in order to analyse the relevance and need to use structural simulations for the estimation of the press-fitted microstructure, a simplified approach consisting of rigid fiber movement with permitted fiber-to-fiber interpenetration was reproduced in the reference bundles to replicate press-fitting without simulating the fiber deformation. This approach proves to be able to well estimate the decrease in bundle porosity as the degree of press-fitting increases. However, when compared the permeabilities estimated with this approach against the permeabilities of deformed microstructures, the errors committed by the simplified approach are progressively bigger as the press-fitting degree increases, up to errors of more than 60% in the main flow direction at a press-fitting level of 15%. This proves the need to consider the structural deformation of the fibers for a correct estimation of permeability at all the degrees of press-fitting. However, when interested in evaluating only low degrees of press-fitting, permeability approximation error of the interpenetrated fibers approach could be considered acceptable. Indeed, at a press-fitting level of 5%, the error in permeability estimation is between 1 and 16% depending on the bundle configuration and flow direction analyzed.

In addition to press-fitting, fiber deformation caused by blood pressure was studied by applying a constant and uniform pressure load on the fiber surfaces of the press-fitted reference bundle. Since the fluid pressure decreases as the fluid passes through the fiber bundle, it was decided to test the fiber deformability in response to the maximum pressure value present in the oxygenator and corresponding to the oxygenator inlet [17].

However, the application of the pressure load shows a negligible change in geometry with an impact on permeability of less than 1% both in the presence and absence of press-fitting. This suggests that the fiber, despite its deformability, has a mechanical stiffness such that the bundle microstructure is not affected by the blood pressure load. Hence, for the identification of deformed bundle geometry, the pressure load can be neglected, differently from the press-fitting which showed a much greater impact.

As the deformation of the bundle microstructure could lead to a change in the fluid dynamics and thus the pressure itself within the bundle, theoretically a fluid–structure interaction (FSI) simulation would be required to determine the deformed fiber geometry and the acting blood pressure. However, the blood pressure has been proved not to be a mechanical load capable of influencing the structure. Therefore, FSI simulations are not necessary and the approach exploiting sequential structural and fluid dynamics simulations is sufficient for a correct description of the bundle geometry and fluid dynamics.

The effects of bundle press-fitting were investigated in different bundles obtained varying some geometrical parameters such as fiber arrangement (orthogonal or angled), fiber size, and pitch between fibers. Only a few values of these parameters were considered with the only purpose of assessing the impact of these parameters on press-fitting. However, a more detailed investigation of the parameters could reveal further interesting aspects in view of an optimization study of bundle geometry where press-fitting could become a design parameter itself.

In this study, the permeability is the fluid dynamics aspect that was mostly investigated. The permeability of fiber bundle is an essential parameter for macroscopic simulations of the whole oxygenator with a porous medium approach. A correct estimation of the press-fitted bundle permeability can be crucial for the assessment of reliable pressure losses of the blood oxygenator. In addition, as permeability provides information on bundle pressure drop, it might be an early indication of some fluid dynamics aspects in the bundle. Indeed, a decrease in bundle permeability involves higher pressure drops within the oxygenator and within the all extracorporeal circulation leading to a higher risk of blood hemolysis [14]. Furthermore, a higher pressure loss could be associated with greater mixing of the blood inside the bundle, which could have beneficial effects on gas exchanges. The preliminary analysis with water of the local velocities shows a significant difference in velocity values and local fluid dynamics due to bundle press-fitting. However, simulations with blood models could confirm and quantify the presence of positive or negative aspects of press-fitting on local fluid dynamics in terms of flow path-induced blood trauma and gas exchange.
